# *Neisseria gonorrhoeae* Challenge Increases Matrix Metalloproteinase-8 Expression in Fallopian Tube Explants

**DOI:** 10.3389/fcimb.2017.00399

**Published:** 2017-09-06

**Authors:** Natalia E. Juica, Paula I. Rodas, Paula Solar, Paula Borda, Renato Vargas, Cristobal Muñoz, Rodolfo Paredes, Myron Christodoulides, Luis A. Velasquez

**Affiliations:** ^1^Facultad de Medicina, Center for Integrative Medicine and Innovative Science, Universidad Andres Bello Santiago, Chile; ^2^Servicio de Ginecología y Obstetricia, Hospital San José Santiago, Chile; ^3^Servicio de Ginecología y Obstetricia, Clínica Indisa Santiago, Chile; ^4^Facultad de Ecología y Recursos Naturales, Escuela de Medicina Veterinaria, Universidad Andres Bello Santiago, Chile; ^5^Neisseria Research Group, Sir Henry Wellcome Laboratories, Division of Infection, Inflammation and Immunity, University of Southampton Medical School Southampton, United Kingdom

**Keywords:** fallopian tubes, *Neisseria gonorrhoeae*, extracellular matrix, metalloproteinases, mucosal damage

## Abstract

**Background:** *Neisseria gonorrhoeae* (Ngo) is the etiological agent of gonorrhea, a sexually transmitted infection that initially infects the female lower genital tract. In untreated women, the bacteria can ascend to the upper genital reproductive tract and infect the fallopian tube (FTs), which is associated with salpingitis and can lead to impaired FT function and infertility. The extracellular matrix (ECM) plays an important role in cell migration and differentiation in the female genital tract, and some pathogens modify the ECM to establish successful infections. The ECM is regulated by matrix metalloproteinases (MMPs) and tissue inhibitors of metalloproteinases (TIMPs), their endogenous inhibitors; MMP deregulation causes pathological conditions in a variety of tissues.

**Results:** The aim of this work was to analyze the expression and localization of MMP-3, MMP-8, MMP-9, and TIMP-1 in FT explants during Ngo infection using real-time PCR, immunohistochemistry, zymography and ELISA. No significant variations in MMP-3, MMP-9, and TIMP-1 transcript levels were observed. In contrast, a significant increase (*p* < 0.05) was observed for MMP-8 expression and was accompanied by stromal immunoreactivity in infected explants. ELISA results supported these findings and showed that MMP-8 release increased upon gonococcal infection.

**Conclusions:** Our results indicate that gonococcal infection induces increased MMP-8 expression, which might contribute to FT damage during infection.

## Introduction

*Neisseria gonorrhoeae* (Ngo) is a Gram-negative diplococcus and the etiological agent of gonorrhea, a sexually transmitted infection exclusive to humans. In women, localized Ngo infections are frequently asymptomatic. However, in approximately 10–25% of untreated women, an ascending infection can involve the upper genital tract and spread to the endometrium, ovaries, myometrium, parametrium and fallopian tubes (FTs). This process leads to a clinical condition known as pelvic inflammatory disease (PID) (Cates et al., 1990; Stacey et al., 1992; Grodstein and Rothman, 1994). The host response to the gonococcal infection manifests as endometritis, tubal abscess and FT inflammation. The latter is termed salpingitis (Wiesenfeld et al., 2005) and can lead to long-term sequelae such as chronic pelvic pain, tubal damage, and ectopic pregnancy (Timmerman et al., 2005).

FTs are seromuscular organs important for mammalian reproduction and serve as the site of fertilization and early zygote development (Lyons et al., 2006). The FT inner mucosa is a columnar epithelium of ciliated, non-ciliated and secretory cells; when gonococci reach the FTs, the bacteria invade and penetrate the extracellular matrix (ECM) by interacting with non-ciliated cells (Virji, 2009). These interactions damage the ciliated cells and eventually cause epithelial cell detachment and significant tissue damage (Stephens et al., 1987).

The ECM is an intricate network of macromolecules, including collagens, elastin, proteoglycans and glycosaminoglycans (Kielty et al., 2002), which play a key role in cell migration, division and differentiation. Because of its distinctive physical and biochemical properties, the ECM is considered an active structure that functions as more than just an organ scaffold (Järveläinen et al., 2009). The ECM is mainly regulated by matrix metalloproteinases (MMPs), a family of zinc-dependent endopeptidases that can cleave most ECM constituents to regulate the cellular microenvironment and process biologically active molecules (Vu and Werb, 2000; Nagase et al., 2006). MMPs play important roles in reproductive tissue remodeling, including during ovulation, menstruation and cervical dilation during childbirth, and their function is regulated at the transcriptional level through zymogen activation and via direct inhibition by tissue inhibitors of metalloproteinases (TIMPs) (Polette et al., 1994; Alexander et al., 1996; Hulboy et al., 1997; Novaro et al., 2002; Noguchi et al., 2003). Therefore, the balance between MMPs and TIMPs is a critical to tissue stability.

The function of MMPs during the infection process has been examined for *Chlamydia trachomatis* (Ault et al., 2002), *Helicobacter pylori* (McClellan et al., 2006), and *Pseudomonas aeruginosa* (Bergin et al., 2008). A recent report evaluated the expression of MMPs during gonococcal infection in FT epithelial cells (FTECs) and observed significantly increased levels of secreted MMP-9 (Rodas et al., 2017). However, the role of MMPs and TIMPs during Ngo infection is not clear and has not been studied in FT explants. Previous studies indicate that MMP-3, MMP-9, and TIMP-1 might participate in FT remodeling during the menstrual cycle (Diaz et al., 2012), whereas MMP-8 might actively function in other infectious processes, such as *Neisseria meningitides* infection (Schubert-Unkmeir et al., 2010). Therefore, the aim of this work was to analyze the role of these ECM regulators in an established model of FT explant infection with Ngo.

## Materials and methods

### Ethics

All protocols were approved by the ethics and biosafety committee of the *Servicio de Salud Metropolitano Norte* and were in accordance with the ethical standards recommended by the Helsinki Declaration (1975). FTs were obtained from women undergoing surgical sterilization for reasons unrelated to this study, and written informed consent was obtained for each participant. The tissues were collected in collaboration with the *Servicio de Ginecolog*í*a y Obstetricia* of the *Hospital San José*. The patients were fertile, 25–45 years of age, and voluntarily requested surgical sterilization. The patients fulfilled all of the requirements for the surgical procedure and all of the inclusion criteria for the study. Exclusion criteria included previous medical history of a sexually transmitted infection, tubal disease, PID, endometriosis, and the use of hormonal contraceptive methods within 3 months prior to surgery. Only ampullar FT segments were obtained because of surgery concerns.

### Human FT explants

The FTs were processed in the laboratory immediately after removal as previously described (Velasquez et al., 2012). Briefly, after muscle dissection, mucosal strips were cut into 1 cm^2^ segments and cultured in Dulbecco's modified Eagle's medium (DMEM, HyClone, USA) supplemented with 10% (vol/vol) fetal bovine serum (FBS, HyClone, USA) for 24 h. The tissues were then deprived of serum for 12 h prior to Ngo infection.

### Growth conditions for Ngo and FT infection

Ngo strain P9 variant 17 (Pil^+^Opa^+^) has been described previously (Virji and Heckels, 1986) and was used for explant infections (Fernandez et al., 2001; Masey et al., 2003). The strain was grown on GC agar (BD, USA) supplemented with Isovitalex (BD, USA) and cultured at 37°C and 5% (vol/vol) CO_2_. The phenotype was confirmed by colony morphology and Western blotting with the specific monoclonal anti-pilus antibody SM1 (Virji et al., 1989) and the anti-Opa protein antibody B33 (Griffiss et al., 1999). FT explants were infected with 100 μL of Ngo suspension (10^5^ colony forming units (cfu/mL) in DMEM for 12 h. Then, both the supernatants and explants tissues were used for reverse transcription-polymerase chain reaction (RT-PCR), immunohistochemistry, zymography and ELISA. Each control and infected explant was derived from the same patient FTs, and eight different patient samples were used. Control explants were grown only in medium.

### RT-PCR for MMP-3, MMP-8, MMP-9, and TIMP-1

Total RNA was purified 12 h post-infection using TRIzol (Invitrogen, USA) according to the manufacturer's protocol. Genomic DNA was removed with RNase-free DNase (Invitrogen, USA). The RNA yield, purity and concentration were determined by spectrophotometry, and RNA integrity was analyzed by electrophoresis using a 1% (wt/vol) agarose gel (Invitrogen, USA). Reverse transcriptase (Invitrogen, USA) was used to convert 1 μg of RNA to cDNA according to the manufacturer's protocol.

Real-time PCR experiments were performed using an Mxpro3000P instrument (Agilent, USA) using specific primers for MMP-3, MMP-8, MMP-9 and TIMP-1 (QIAGEN, USA; cat number PPH0023F, PPH00908C, PPH00152E, and PPH00771C, respectively) with programs recommended by the manufacturer. The specificity of the amplified fragments was confirmed with a dissociation curve after the amplification program.

Ct values were used to calculate the fold change in gene expression between infected and control explants using the 2^−ΔΔCt^ method (Livak and Schmittgen, 2008; Bustin et al., 2009). Statistical analysis was performed using the Wilcoxon test, and differences were considered statistically significant at *p* < 0.05. PCR experiments were performed 8 times.

### Immunohistochemistry

Immunohistochemistry experiments were performed on four samples (*n* = 4) of infected and uninfected FTs explants, and specific anti-human antibodies against MMP-3 (1:250, AbD Serotec, USA), MMP-8 (1:200 dilution, AbD Serotec, USA), MMP-9 (1:100 dilution, AbD Serotec, USA), and TIMP-1 (1:20 dilution, AbD Serotec, USA) were used. Briefly, FT explants were cut into 5 mm^2^ pieces and fixed in 4% (wt/vol) paraformaldehyde for 2 h. Pieces were then placed in a saccharose gradient (5–20%) and embedded in paraffin. Fixed samples on slides were rehydrated and treated with 1.5% hydrogen peroxide in distilled water for 30 min to block endogenous peroxidase activity. The tissues were washed with phosphate-buffered saline with 0.1% (vol/vol) Tween (PBS-Tween) and incubated with non-immune serum for 60 min. The primary antibody was added, the samples were stored at 4°C for 16 h, and then the Histostain®-SP kit (Invitrogen, USA) was used according to the manufacturer's instructions. The negative control explants were not treated with primary antibody. Tissue sections were counterstained with Harris hematoxylin (Sigma, USA), air-dried and mounted. The background was subtracted using ImageJ software (NIH, USA), and immunoreactivity was analyzed using a double-blind, semi-quantitative assessment using the following scores: +++ denotes intense staining; ++ denotes moderate staining, and + denotes minimal staining.

### MMP-9 analysis

For MMP-9 analysis, 25 μg of total protein from FT explant supernatants was separated by electrophoresis on acrylamide/bisacrylamide gels copolymerized with gelatin (Hu and Beeton, 2010). After electrophoresis was complete, the gels were incubated in renaturation buffer for 15 min and then incubated with development buffer for 24 h at 37°C with gentle agitation. After the gels were washed, they were stained with 0.5% Coomassie blue in methanol:acetic acid:water (4:1:5) for 1 h at RT with gentle agitation. Finally, destaining was performed using methanol:acetic acid:water (4:1:5) for 1 h at RT until clear bands were observed. The presence of MMPs was determined based on the corresponding molecular weights of the visualized proteolytic bands and compared with those of the corresponding MMP controls. Enzyme activities in the gel were quantified with respect to band intensity using ImageJ software (NIH, USA). Seven different supernatants from infected and control explants were evaluated in triplicate.

### MMP-3, MMP-9, and TIMP-1 expression

Secreted MMP-8 (Abcam, USA), MMP-3 (R&D, USA) and TIMP-1 (Thermo, USA) were analyzed by sandwich immunoassay according to manufacturer's protocols using supernatants from infected and uninfected explants. For MMP-3 and TIMP-1, primary antibody coating was performed overnight at RT. After 2 h of plate blocking, the supernatants were incubated for 2 h at RT. The plates were washed four times and then incubated with the detection antibody for 2 h. Streptavidin-HRP substrate was added for 20 min. After the addition of stop solution, readings were performed at 450 and 570 nm in a Synergi H1 Biotek spectrophotometer.

For the MMP-8 ELISA, the same steps were performed except the primary antibody was already coated on the plate, and the samples were incubated overnight at RT.

Statistical analysis was performed using the Wilcoxon test, and differences were considered statistically significant at *p* < 0.05. For ELISA analysis, four different samples were evaluated in triplicate.

## Results

### Ngo infection induces an increase in MMP-8 but not MMP-3, MMP-9, or TIMP-1 transcripts

**Figure 1:**
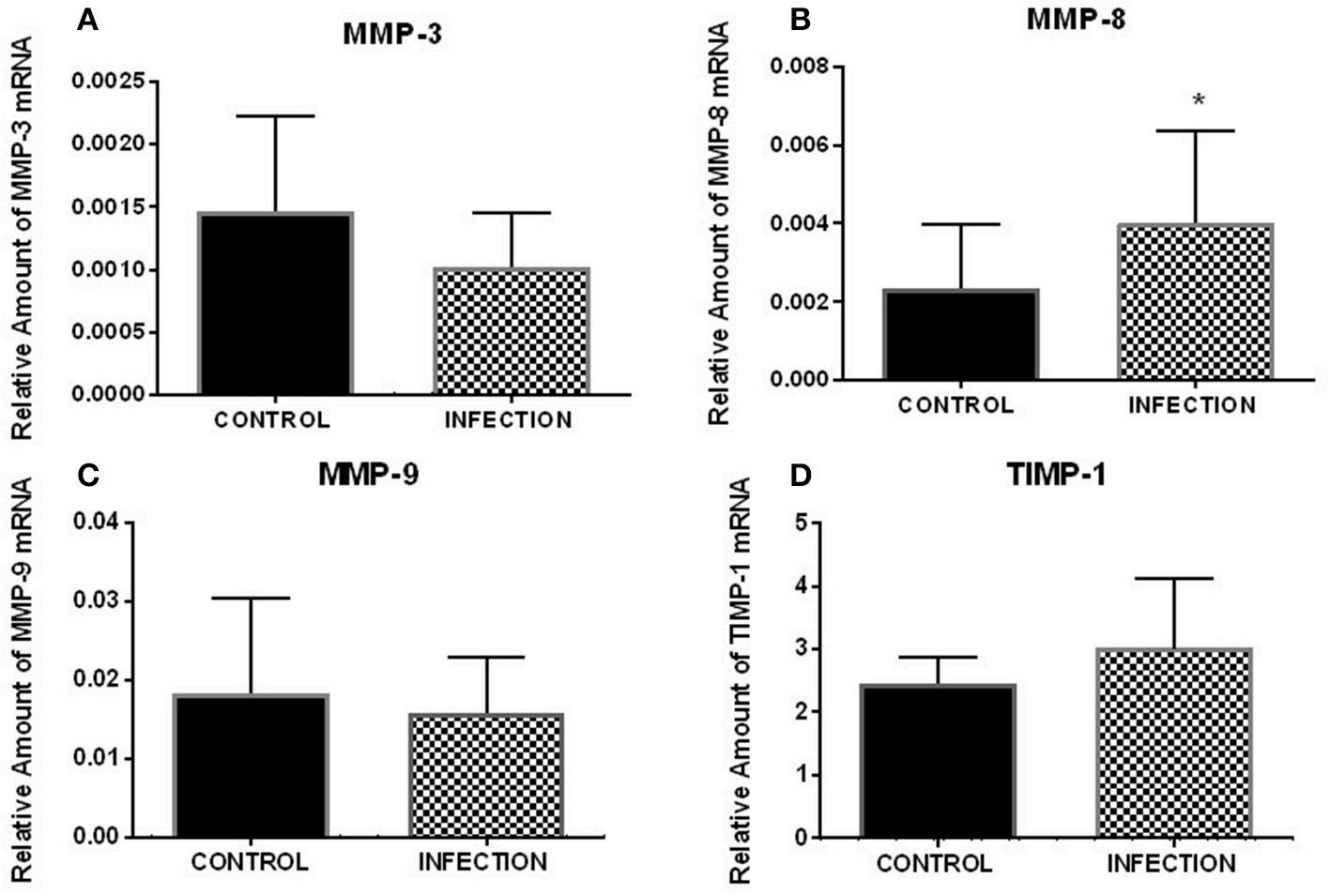
Relative expression of analyzed genes after FT explant infection with Ngo. Relative expression of MMP-3 **(A)**, MMP-8 **(B)**, MMP-9 **(C)**, and TIMP-1 **(D)** after 12 h of infection (*n* = 8). The results show a significant variation in MMP-8 (*p* < 0.05). Each bar represents the mean ± SEM. ^*^Indicates a significant change.

The mRNA levels of MMP-3, MMP-8, MMP-9, and TIMP-1 were analyzed 12 h after FT explant infection with Ngo strain P9, variant 17 (Pil^+^Opa^+^). Ngo induced a significant increase (*p* < 0.05) in MMP-8 expression compared with that in uninfected explants (Figure 1). However, no significant difference (*p* > 0.05) in MMP-3, MMP-9, or TIMP-1 transcripts was observed between Ngo-infected and uninfected explants. All of the data were normalized to the RPL13A housekeeping gene.

### MMPs localization in uninfected and infected explants

**Figure 2:**
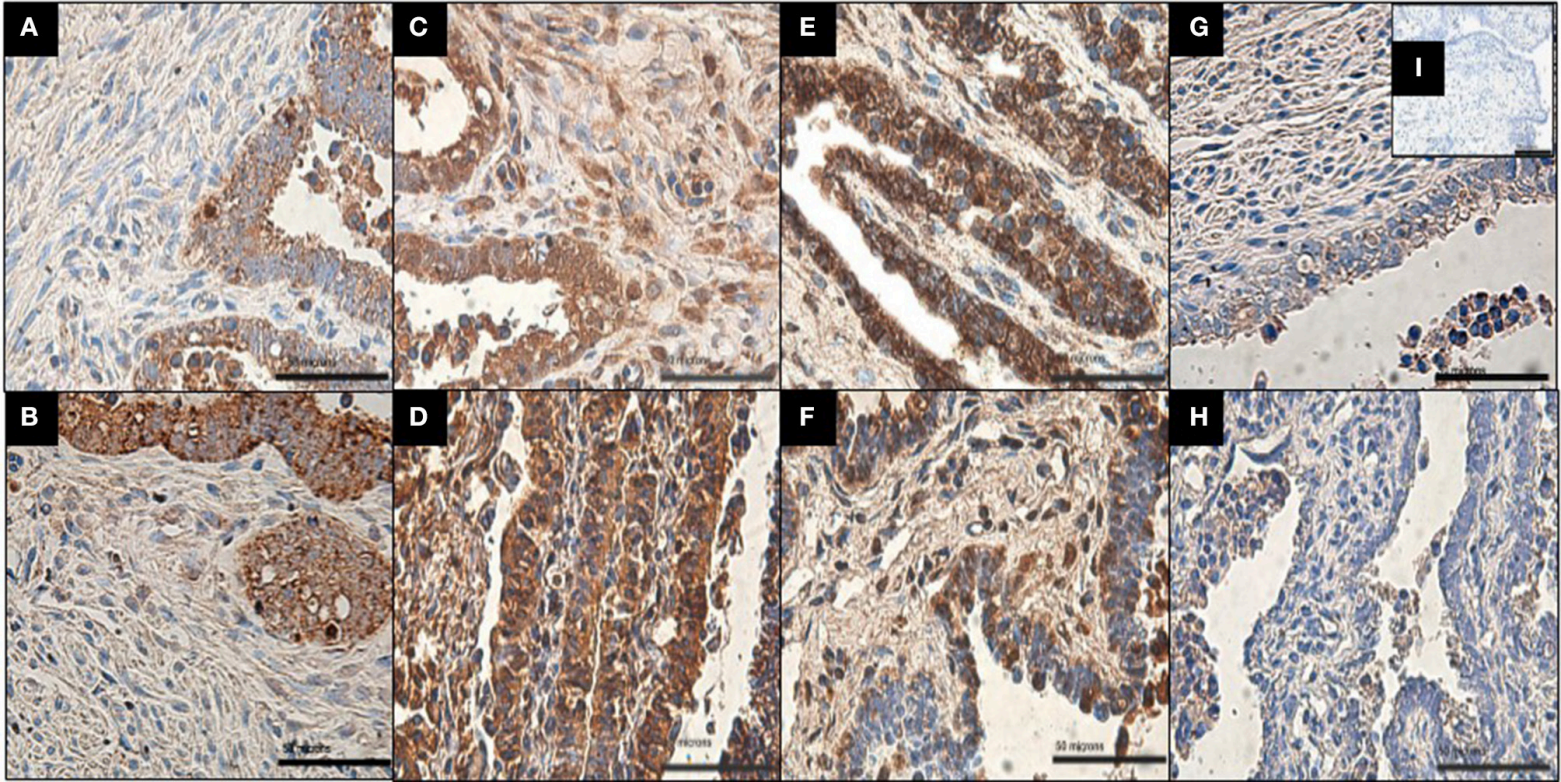
Immunolocalization of MMP-9, MMP-3, MMP-8, and TIMP-1 in infected and uninfected explants. **(A,C,E,G)** Show uninfected explant expression of MMP-9, MMP-3, MMP-8, and TIMP-1, respectively. **(B,D,F,H)** Show infected explant expression of MMP-9, MMP-3, MMP-8, and TIMP-1. All of the proteins were detected in the stroma and epithelial cells in both conditions. Note that MMP-8 shows markedly increased immunoreactivity in stroma after Ngo challenge and less immunoreactivity in epithelial cells than in uninfected explants (*n* = 4). Scale bar = 50 μm. Negative control **(I)** shows no staining.

The localization of MMP-3, MMP-8, MMP-9, and TIMP-1 proteins on infected and uninfected FT explant sections was assessed by immunohistochemistry. All of the proteins were detected in the stroma and epithelium of infected and uninfected explants (Figure 2). However, MMP-8 exhibited specific localization in the stroma of infected explants, in contrast to control explants, which showed predominantly epithelial expression of the protease (Table 1).

**Table 1:** Immunoreactivity analysis of FT explants infected with Ngo.

	**Uninfected**		**Infected**	
**Protein**	**Epithelium**	**Stroma**	**Epithelium**	**Stroma**
MMP-9	^++^	^++^	^+++^	^++^
MMP-3	^++^	^+^	^++^	^+^
MMP-8	^+++^	^++^	^+^	^++^
TIMP-1	^+^	^+^	^+^	^+^

+++ , intense staining;

++ , moderate staining; and

+ *, minimal staining*.

### MMPs expression

**Figure 3:**
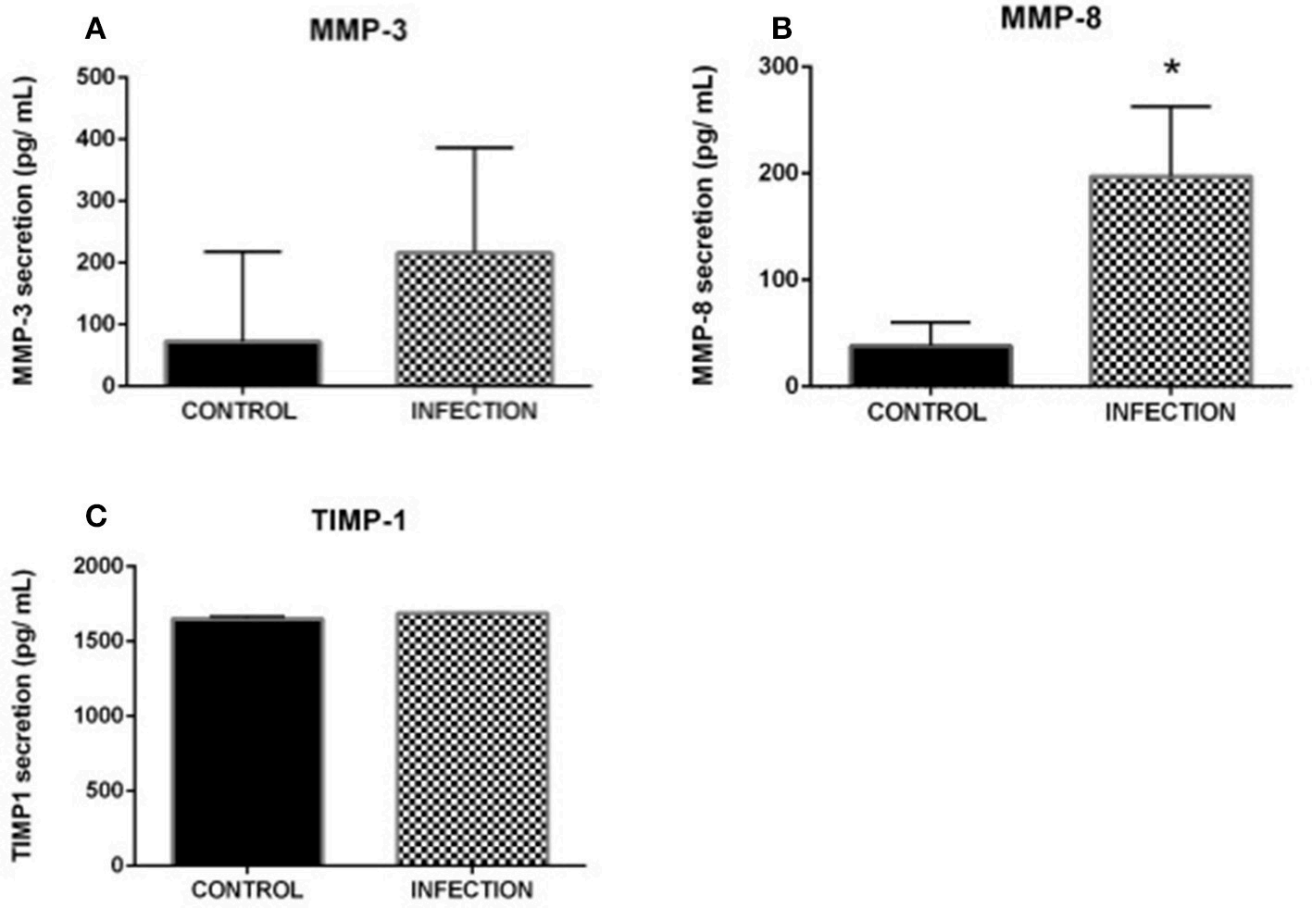
Evaluation of MMP-3 **(A)**, MMP-8 **(B)**, and TIMP-1 **(C)** protein expression upon gonococcal infection. Supernatant from control and infected explants was evaluated with sandwich immunoassays to evaluate the secretion of MMP-3, MMP-8 and TIMP-1. A significant increase in MMP-8 secretion was observed from infected explants compared with that in the control (*p* < 0.05); the other evaluated proteins showed no significant variation. Each sample was assayed in duplicate, and four different patient samples were used. Each bar represents the mean ± SEM. ^*^Indicates a significant change.

Supernatants from control and infected explants were evaluated by immunoassay specifically for MMP-8, MMP-3, and TIMP-1 secretion. A significant increase in MMP-8 was observed in supernatants from infected explants compared with that in uninfected controls (Wilcoxon test, *p* < 0.05; Figure 3). MMP-3 and TIMP-1 were not significantly different between uninfected and infected explants.

**Figure 4:**
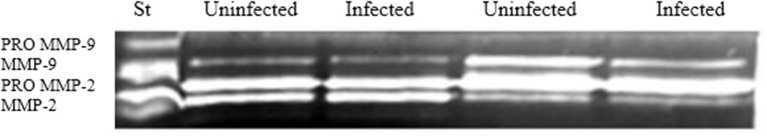
MMP-9 gelatinase activity. Supernatants from control and infected explants were analyzed by gelatin zymography to evaluate MMP-9 activity (*n* = 7). No significant changes were observed after densitometric analysis.

Zymography was performed to evaluate the gelatinase activity of MMP-9 All of the samples exhibited gelatinase activity, as shown in Figure 4. Densitometric analysis indicated that no significant differences were detected in MMP-9 activity between infected and uninfected explants (data not shown).

## Discussion

Ngo infection is characterized by the detachment of epithelial cells from the basal lamina, which can result in tubal damage and infertility (Westrom and Wolner-Hanssen, 1993). These processes occur due to ECM modification caused by inflammation in the FTs caused by the repair process or immune cell recruitment to the site of infection. In this study, we evaluated expression changes of the ECM regulators MMP-3, MMP-8, MMP-9, and TIMP-1 in FT explants infected with Ngo.

Under normal conditions, all of the proteins were expressed in FT explants. Although expression of these MMPs has been described in the ovaries and uterus (Currt and Osteen, 2001), our study is the first to demonstrate MMP-3, MMP-8, MMP-9, and TIMP-1 expression in ampullar FT organ culture.

Moreover, real-time PCR revealed a significant increase in MMP-8 transcript levels for infected explants compared with those in uninfected explants (*p* < 0.05), which was accompanied with a significant increase in MMP-8 secretion.

MMP-8 is a neutrophil collagenase, but it can also be expressed by fibroblast and endothelial cells (Danielsen et al., 2011). In the case of ampullar FTs explants this was also corroborated by immunohistochemistry, were MMP-8 was found to be also located in the stroma (Figure 2) that is mainly composed by fibroblast-mesenchymal cells (Wang et al., 2015). In FTs connective tissue, fibers of Collagen I and II are expressed (Schultka et al., 1989). Considering that collagen types I, II and II are the main substrates for MMP-8 activity and that this MMP has been reported to participate in tissue remodeling processes during inflammatory conditions (Warner et al., 2004), an increase in transcript levels and secretion of this protease may lead to a greater collagen degradation which could contribute to tissue instability and eventually epithelial cell detachment, as observed in gonococcal infection (McGee et al., 1981).

It has also been reported that MMP-8 is capable to mediate occludin degradation (Nagase et al., 2006) which may contribute to epithelial line disruption in the FTs indicating that MMP-8 could play a key role in gonococcal pathogenesis.

Besides ECM components, MMPs can act on other substrates such as proinflammatory cytokines to mediate their direct activation (Rodriguez et al., 2010). Cytokines play an essential role in the course of Ngo infection and are produced by FTs epithelial cells (Morales et al., 2006); thus significant cytokine production would be anticipated when gonococcal bacteria reach the subepithelial tissue, followed by the recruitment of immune cells (neutrophils) to the infection site. Indeed, TNF-α, IL-1β, IL-6, and IL-8 production has been observed in FTs explants infected with Ngo (McGee et al., 1992), and an increased level of proinflammatory cytokines has been associated with a high level of MMPs (Mauviel, 1993). In this way, bibliographics and bioinformatics reports indicates that MMP-8 cleaves IL-8 generating a more active chemokine, enhancing neutrophil chemotaxis and therefore increasing MMP-8 secretion (Van den Steen et al., 2003; Van Lint and Libert, 2006; Thirkettle et al., 2013).

No significant differences in MMP-9 and MMP-3 transcript levels (*p* > 0.05) and protein immunoreactivity were observed in infected and uninfected explants. However, MMPs can be regulated at the post-transcriptional level (Nagase et al., 2006); for example, MMPs may be pre-synthesized and activated after secretion in response to infection. Gelatin zymography was use to evaluate MMP-9 and results revealed no significant differences in the activity of this MMPs between infected and uninfected explants (Figure 4). A recent work of our group indicates that MMP-8 and MMP-3 were not present in primary culture of fallopian tube epithelial cells when infected with Ngo, however these MMPs were present in FTs explants, which indicate that epithelial-stroma interaction may have an important role during gonococcal infection (Rodas et al., 2017).

## Ethics statement

All protocols were approved by the ethics and biosafety committee of the Servicio de Salud Metropolitano Norte and were in accordance with the ethical standards recommended by the Helsinki Declaration (1975). FTs were obtained from women undergoing surgical sterilization for reasons unrelated to this study, and written informed consent was obtained for each participant. The tissues were collected in collaboration with the Servicio de Ginecología y Obstetricia of the Hospital San José. Patients were fertile, 25 to 45 years of age, and voluntarily requested surgical sterilization. The patients fulfilled all requirements for the surgical procedure and all criteria for inclusion in the study. Exclusion criteria included previous medical history of a sexually transmitted infection, tubal disease, PID, endometriosis, and the use of hormonal contraceptive methods within three months prior to surgery. For this work, only ampullar FTs segments were obtained.

## Conclusions

Our work is the first report about MMPs expression in the FTs under infection process. This founding was accompanied with a significant increase of MMP-8 transcript level and protein secretion on infected ampullar FT explants when compared to uninfected controls. These results must be complemented with evaluation in other study models and even considering that there is no available *in vivo* model to evaluate the effects of gonococcal infection, this piece of information will be important for the generation of new strategies to evaluate the role of MMPs on gonococcal infection. Understanding the role of MMPs in gonococcal infection will increase our knowledge about pathogenesis of salpingitis and contribute to treatment strategies for gonococcal infection.

## Author contributions

NJ performed fallopian tube processing, Real time PCR, zymography assays, infection assays and participate on IHQ assays. PR supervised ELISA, zymography assays and bacterial cell culture. PS support real time PCR assays and statistical analysis. PB and RV performed the laparoscopic operations to collect the fallopian tubes. CM and RP performed IHQ assays and. MC collaborate and help to draft the manuscript. LV conceived and designs the study, also coordinates and helped to draft the manuscript. All authors read and approved the final manuscript.

### Conflict of interest statement

The authors declare that the research was conducted in the absence of any commercial or financial relationships that could be construed as a potential conflict of interest.

## Abbreviations

Ngo: *Neisseria gonorrhoeae*
PID: Pelvic Inflammatory disease
FTs: Fallopian tubes
ECM: Extracellular Matrix
MMPs: Matrix Metalloproteinases
TIMPs: Tissue inhibitors of Metalloproteinases.
